# Mutations in the Human *naked cuticle* Homolog *NKD1* Found in Colorectal Cancer Alter Wnt/Dvl/β-Catenin Signaling

**DOI:** 10.1371/journal.pone.0007982

**Published:** 2009-11-24

**Authors:** Jianhui Guo, Tolga Cagatay, Guangjin Zhou, Chih-Chiang Chan, Shelby Blythe, Kaye Suyama, Li Zheng, Kaifeng Pan, Chiping Qian, Richard Hamelin, Stephen N. Thibodeau, Peter S. Klein, Keith A. Wharton, Wanguo Liu

**Affiliations:** 1 Department of Genetics, Louisiana State University Health Sciences Center/Stanley S. Scott Cancer Center, New Orleans, Louisiana, United States of America; 2 Laboratory of Molecular Pathology, Department of Pathology, and Department of Molecular Biology, Simmons Comprehensive Cancer Center, UT Southwestern Medical Center, Dallas, Texas, United States of America; 3 Department of Laboratory Medicine and Pathology, Mayo Clinic/Mayo Clinic College of Medicine, Rochester, Minnesota, United States of America; 4 Department of Medicine, University of Pennsylvania, Philadelphia, Pennsylvania, United States of America; 5 Department of Developmental Biology, Stanford University School of Medicine, Stanford, California, United States of America; 6 INSERM U938 Equipe Microsatellites et Cancers, Paris, France; 7 Howard Hughes Medical Institute, Stanford University School of Medicine, Stanford, California, United States of America; 8 Department of Cell and Developmental Biology, University of Pennsylvania, Philadelphia, Pennsylvania, United States of America; University of Birmingham, United Kingdom

## Abstract

**Background:**

Mutation of Wnt signal antagonists Apc or Axin activates β-catenin signaling in many cancers including the majority of human colorectal adenocarcinomas. The phenotype of *apc* or *axin* mutation in the fruit fly *Drosophila melanogaster* is strikingly similar to that caused by mutation in the segment-polarity gene, *naked cuticle (nkd)*. Nkd inhibits Wnt signaling by binding to the Dishevelled (Dsh/Dvl) family of scaffold proteins that link Wnt receptor activation to β-catenin accumulation and TCF-dependent transcription, but human *NKD* genes have yet to be directly implicated in cancer.

**Methodology/Principal Findings:**

We identify for the first time mutations in *NKD1* - one of two human *nkd* homologs - in a subset of DNA mismatch repair-deficient colorectal tumors that are not known to harbor mutations in other Wnt-pathway genes. The mutant Nkd1 proteins are defective at inhibiting Wnt signaling; in addition, the mutant Nkd1 proteins stabilize β-catenin and promote cell proliferation, in part due to a reduced ability of each mutant Nkd1 protein to bind and destabilize Dvl proteins.

**Conclusions/Significance:**

Our data raise the hypothesis that specific *NKD1* mutations promote Wnt-dependent tumorigenesis in a subset of DNA mismatch-repair-deficient colorectal adenocarcinomas and possibly other Wnt-signal driven human cancers.

## Introduction

Activation of “canonical” Wnt/β-catenin signaling in nearly all human colorectal adenocarcinomas (CRC) makes the Wnt pathway a promising yet untapped therapeutic target [1]. The prevailing paradigm for canonical Wnt signaling was deduced in part through elegant developmental studies of the fruit fly *Drosophila melanogaster* and the amphibian *Xenopus laevis*: Absent the Wnt signal, a “destruction complex” composed of the proteins Apc, Axin, GSK3β, and CK1 phosphorylates β-catenin, leading to β-catenin ubiquitination and proteasomal degradation [2]. Binding of Wnt ligands to Frizzled/Lrp coreceptors activates the scaffold protein Dishevelled (Dsh; Dvl1, Dvl2, Dvl3 in mammals), leading to sequestration and degradation of Axin, which allows β-catenin to accumulate, enter the nucleus, and bind TCF transcription factors to regulate target genes [2].

A majority (60–85%) of human CRC exhibit activated canonical Wnt signaling due to truncating mutations in *APC* that stabilize β-catenin [3]. Alternatively, mutations in β-catenin *(CTNNB1)* that block phosphorylation and degradation are found in some CRC that lack *APC* mutation [4]. CRCs display at least two types of genomic instability: chromosomal instability (CIN) associated with mutant Apc and p53 and giving rise to aneuploidy, and microsatellite-instability (MSI) caused by defective DNA mismatch repair (MMR) and resulting in mutations in simple sequence repeats (SSR) throughout the genome [5], [6]. Mutation of SSRs in the coding or splice junction regions of key regulatory genes can create point mutant or truncated proteins that promote cancer progression; indeed, MMR deficiency and MSI are characteristic of tumors in patients with hereditary nonpolyposis colorectal cancer syndrome {HNPCC; a.k.a. Lynch Syndrome (OMIM 120435)} and of 13–17% of sporadic CRC [7]. *APC* mutations are prevalent in CIN-CRC [3], but the Wnt pathway gene mutation spectrum in MSI-CRC is less well characterized, with mutations in the Axin homolog *AXIN2* and the TCF-family transcription factor *TCF7L2* identified in ∼25% and ∼35% of MSI-CRC, respectively [8], [9]. *APC* mutation is less frequent in MSI-CRC than in CIN-CRC [10], [11], while activating mutations in *CTNNB1*, though widespread throughout the spectrum of human cancer, are rare in MSI-CRC [11]. These data suggest that additional mechanisms activate Wnt/β-catenin signaling in MSI-CRC.

**Figure 1:**
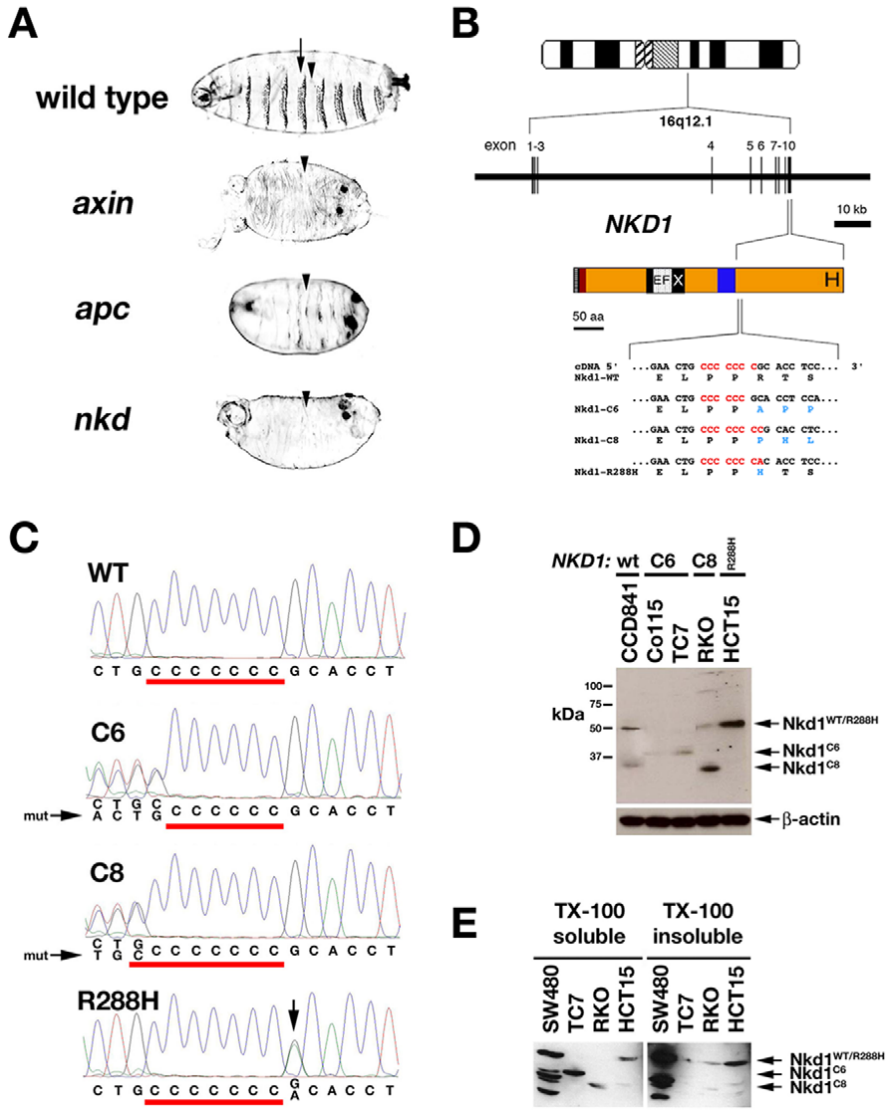
*Nkd* mutations in fly and human. (A) Wild-type, *axin, apc*, and *nkd Drosophila* cuticles. Wild type has alternating denticle bands (arrow) and naked cuticle (arrowhead), with each mutant lacking denticle bands. (B) *NKD1* locus has 10 exons. Nkd1 schematic (orange) includes N-terminal myristoylation, EFX, 30aa (blue), and carboxy-terminal His-rich motifs. Exon 10 sequences around poly-(C) tracts (red) above native (black) and mutant (blue) residues are shown. (C) *NKD1* electropherograms showing wild-type (WT) poly-(C)_7_, cell line TC7 with C-deletion (C6), cell line RKO with C-insertion (C8), and cell line HCT15 with G>A mutation (arrow) 3′ of poly-(C)_7_. (D, E) α-Nkd1 blots of whole cell extracts (D) and Triton X-100 soluble and insoluble fractions (E) from cell lines with indicated *NKD1* mutation. Arrows designate Nkd1 proteins. β-actin is loading control in D. CCD841 has full length Nkd1, with a minor degradation product at ∼35 kDa also seen with transfected *NKD1* (e.g. Fig. 1E, 5C), whereas SW480 with more abundant but wild-type Nkd1 has several degradation products.

The Naked cuticle (Nkd) protein family attenuates canonical Wnt signaling by binding and possibly destabilizing Dsh/Dvl proteins [12]–[17]. *Drosophila nkd* mutants develop lethal segmentation defects very similar to those seen in *apc* or *axin* mutants [12], [18], [19] (Fig. 1A). We therefore hypothesized that alteration of *nkd* gene activity in mammals might activate Wnt signaling and cause cancer. Here we identify novel mutations in the human *NKD1* gene in MSI-CRC that alter Wnt signaling and reduce Nkd/Dsh interactions. Our data suggest that specific *NKD1* mutations alter Wnt/β-catenin signaling in a minority of MSI-CRC as well as possibly in other β-catenin signal-dependent tumors in which mutations in the known Wnt regulators are infrequent.

## Results

### *NKD1* mutations in colorectal adenocarcinoma

We identified three different *NKD1* exon 10 coding region mutations in 5/11 CRC cell lines and 2/40 sporadic CRC tumors with MSI (Table 1), but no *NKD1* coding region or splice junction mutations in 5/5 CRC cell lines and 50/50 tumors without MSI. Two mutations, either a deoxycytidine (C) deletion or insertion due to polymerase slippage within an exon-10 poly-(C)_7_ tract, result in the synthesis of truncated proteins of 345 or 298 amino acids (aa)(Fig. 1B,C). A (C)_7_-adjacent missense mutation (G>A) converts Arg-288, conserved in Nkd2, to His (Fig. 1B,C). *NKD1* mutations were not detected in 32/40 MSI-CRC tumors that harbor mutations in other Wnt-pathway genes including *APC*, *CTNNB1*, *AXIN2*, and *TCF7L2*, but were found in 2 of the remaining 8/40 tumors without lesions in these known Wnt-pathway genes (p = 0.036, one-tailed Fisher's exact test) (Table 1; see Materials and Methods). These data indicate a mutual exclusivity among mutations in *NKD1* and other Wnt-pathway genes in our cohort of MSI-CRC tumor samples, suggesting that the *NKD1* mutations are of pathological significance.

**Table 1:** Wnt-pathway genetic lesions in MSI colon tumors and cell lines.

*Tumor ID*	*NKD1*	*AXIN2*	*TCF7L2*	*CTNNB1*	*APC*
CT1	−	1994ins G	A8/A9	−	−
CT2	−	−	A8/A9	−	−
CT3	−	−	A8/A9	−	−
CT4	−	1994ins G	−	−	−
CT5	−	−	A8/A9	−	T
CT6	−	−	−	−	−
CT7	−	−	A8/A9	−	T
CT8	872del C	−	−	−	−
CT9	−	−	−	M	−
CT10	−	−	A8/A9	−	−
CT11	−	−	A8/A9	−	−
CT12	−	−	−	−	T
CT13	−	1994ins G	−	−	−
CT14	−	−	−	−	−
CT15	−	2023del C	−	−	−
CT16	−	−	−	−	T
CT17	−	1994ins G	A8/A9	−	−
CT18	−	−	A8/A9	−	−
CT19	−	2011del C	−	−	−
CT20	−	−	−	−	−
CT21	−	1925ins A	−	−	−
CT22	−	−	−	M	−
CT23	−	−	−	M	−
CT24	−	−	−	−	−
CT25	−	−	A8/A9	−	−
CT26	−	−	A8/A9	−	−
CT27	872del C	−	−	−	−
CT28	−	−	A8/A9	−	−
CT29	−	−	A8/A9	−	−
CT30	−	1994del G	−	−	−
CT31	−	−	A8/A9	−	−
CT32	−	−	−	M	−
CT33	−	1994del G	−	−	−
CT34	−	−	A8/A9	−	−
CT35	−	−	−	M	−
CT36	−	−	−	−	−
CT37	−	−	A8/A9	−	−
CT38	−	−	A8/A9	−	−
CT39	−	−	−	−	−
CT40	−	−	A8/A9	−	−

*Cell line*	*NKD1*	*AXIN2*	*TCF7L2*	*CTNNB1*	*APC*
Co115	872del C	−	−	−	−
LS174T	−	−	A8/A9	+	−
LoVo	−	−	A8/−	−	+
TC71	−	−	A8/−	−	+
HCT15	873G>A	−	−	−	+
HCT116	−	1994del G	−	+	−
TC7	872del C	−	A8/A9	−	+
SW48	−	−	−	+	−
RKO	872ins C	−	−	−	−
HCT8	−	−	−	−	−
LS411	872ins C	−	−	−	+

*NKD1, AXIN2, TCF7L2, CTNNB1*, and *APC* gene mutation status in 40 MSI-CRC tumors (top) and 11 cell lines (bottom). Key: −, no lesion; ins, nucleotide insertion; del, nucleotide deletion; /−, allelic deletion. For *NKD1* and *AXIN2*, mutation in indicated nucleotide is designated, while for *TCF7L2* the status of the poly(A) tract (A8–wild-type; A9–mutant) is designated. *CTNNB1* exon-3 was screened for activating mutations (M). For *APC*, tumors positive for protein-truncation (T) are indicated [45]. Presence (+) or absence (−) of *APC* and *CTNNB1* lesions in each cell line is as described [48].

Both the colonic epithelial cell line CCD841 and the CRC cell line SW480, the latter with a C>T mutation at *APC* codon #1338 [20], encode a wild-type Nkd1 (470 aa) of 53.2 kDa that migrates at ∼50 kDa on western blot (Fig. 1D, E). Cell lines Co115 and TC7, each with the (C)_6_ mutation, have a ∼39 kDa band that corresponds to the 38.1 kDa Nkd1^C6^, while cell line RKO, with the (C)_8_ mutation, has a ∼33 kDa band that corresponds to the 33.4 kDa Nkd1^C8^; all three cell lines with truncated Nkd1 also have less abundant full-length Nkd1, suggesting that the wild-type *NKD1* locus does not undergo loss-of-heterozygosity (Fig. 1D). By western blot with Nkd1 antibodies we are unable to distinguish wild-type from mutant Nkd1 in cell line HCT15, which harbors *NKD1^R288H^* (Fig. 1D).

Nkd proteins are membrane-associated, the mammalian Nkds by virtue of N-terminal myristoylation [14], [21], [22]. The truncated Nkd1 proteins have a reduced affinity for membranes compared to full-length Nkd1 as inferred by Triton X-100 solubility: 95% of Nkd^C6^ or Nkd^C8^ from mutant cell lines is TX-100 soluble, whereas 0–26% of full-length Nkd1 from all cell lines is TX-100 soluble (Fig. 1E).

### Mutant Nkd1 proteins are defective at inhibiting Wnt/Dvl signaling

**Figure 2:**
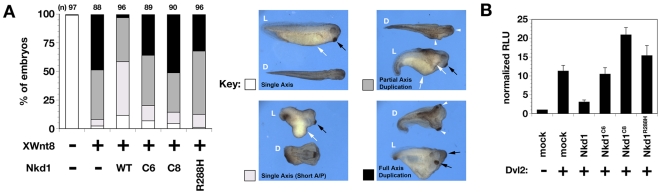
Wild-type Nkd1 but not mutant Nkd1 inhibits Wnt/β-catenin signaling. (A) % *Xenopus* embryos with indicated axis phenotype after injection of *XWnt8*+/−wild-type or mutant *NKD1*. (n) = # embryos injected. Panels at right show representative lateral (L) and dorsal (D) views of embryos scored as single axis (white), short A/P axis (light gray), partial axis duplication (dark gray), and full axis duplication (black) according to the Materials and Methods. In embryos with single axis in the left panels, note trunk (white arrow) and cement gland (black arrow). Arrowheads designate each axis in embryos with axis duplications (right panels). Embryos with partial axis duplication have duplicated trunk tissue but a single cement gland, while embryos with full axis duplication have duplicated trunk tissues and cement glands. (B) Normalized TOPflash luciferase activity (RLU) in HEK-293 cells transfected with reporter +/−Dvl2 +/− indicated Nkd1 construct.

Mouse Nkd1 can inhibit axis duplication induced by ectopic Wnt signaling in *Xenopus* embryos [16]. As shown in Fig. 2A, >90% of *Xenopus* embryos injected with *XWnt8* mRNA developed partial-to-complete axis duplication; consistent with Nkd1's activity as a Wnt antagonist, wild-type human *NKD1* mRNA co-injection reduced axis duplication frequency to 42%, with only 3% complete duplications. In contrast, co-injection of each mutant human *NKD1* resulted in axis duplication frequencies similar to that observed with *XWnt8* injection alone (Fig. 2A). As in cell lines, misexpressed Nkd1^C6^ and Nkd1^C8^ were more abundant than wild-type Nkd1 or Nkd1^R288H^ by western blot (not shown). In agreement with the *Xenopus* results, wild-type Nkd1, but none of the three mutants, suppressed Dvl-induced activation of the TCF-reporter TOPflash in HEK-293 cells (Fig. 2B). Expression of mutant Nkd1 alone slightly increased basal TOPflash activity and had no effect on endogenous *Xenopus* axis formation (not shown), indicating that the effect of Nkd1, like that of *nkd* in the fly, depends on Wnt signaling [23].

### *NKD1* mutations stabilize β-catenin and promote cell proliferation

**Figure 3:**
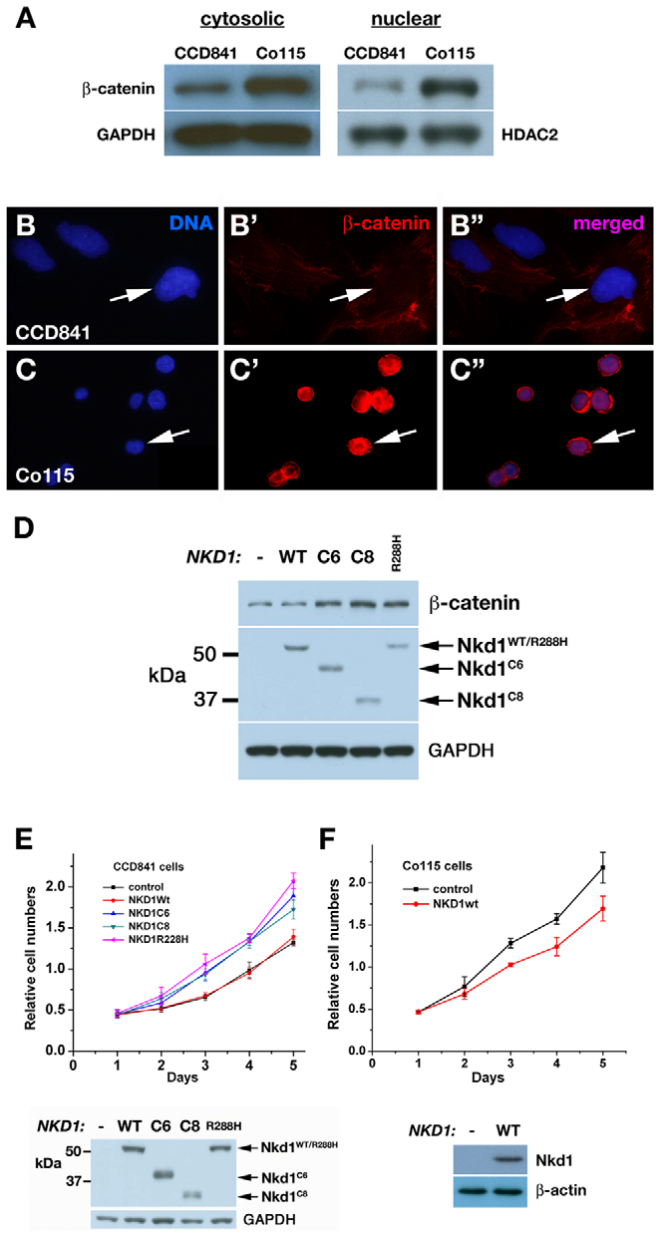
Effect of *NKD1*-mutations on β-catenin and cell proliferation. (A) Western blot of cytosolic and nuclear extracts of cells with wild-type (CCD841) or mutant (Co115) *NKD1*. GAPDH and HDAC2 were probed as loading controls. (B–B″, C–C″) β-catenin (red) and DNA (blue) distribution in CCD841 cells (B–B″) and Co115 (C–C″) cells. Arrows designate nuclei. Merged images in B″ and C″. (D) Western blot of cytosolic extracts of HEK-293 cells transfected with *lacZ* control (-), wild type Nkd1 (WT), or indicated mutant Nkd1 construct, and probed for β-catenin, Nkd1, and loading control GAPDH. Note that each mutant Nkd1 but not wild type Nkd1 increases β-catenin levels. (E) Relative cell number as a function of days post retroviral infection of CCD841 cells with empty vector control, wild type Nkd1, or indicated Nkd1 mutant (p = 0.016, 0.012, and 0.0091 for C6, C8, and R288H mutants as compared to control) (F) Relative cell number as a function of days post retroviral infection of Co115 cells with control or wild-type Nkd1 (p = 0.022). α-Nkd1 western blots of cell extracts, with GAPDH or β-actin loading control, are shown below each plot in E and F.

Consistent with the *NKD1* mutations activating Wnt signaling in CRC, cytoplasmic and nuclear levels of β-catenin are higher in Co115 cells than in CCD841 cells (Fig. 3A). Accordingly, nuclear β-catenin was prominent in Co115 cells but not in CCD841 cells (Fig. 3B, C). HEK-293T cells transfected with Nkd1^C6^, Nkd1^C8^, or Nkd1^R288H^ had higher levels of cytosolic β-catenin than cells transfected with wild-type Nkd1 or a control (Fig. 3D), indicating that each mutant Nkd1 protein can stabilize β-catenin.

Since canonical Wnt signaling promotes cell proliferation [2], we assayed the accumulation of cells uniformly expressing comparable levels of either wild-type or mutant Nkd1. Retroviral expression of each mutant Nkd1 protein in CCD841 cells increased cell numbers compared to wild-type Nkd1 or empty vector control (Fig. 3E). Conversely, expression of wild-type Nkd1 in Co115 cells reduced cell numbers (Fig. 3F). These data indicate that the *NKD1* mutations can promote β-catenin stabilization and colonic cell proliferation.

### Altered subcellular localization and Dvl colocalization of truncated Nkd1

**Figure 4:**
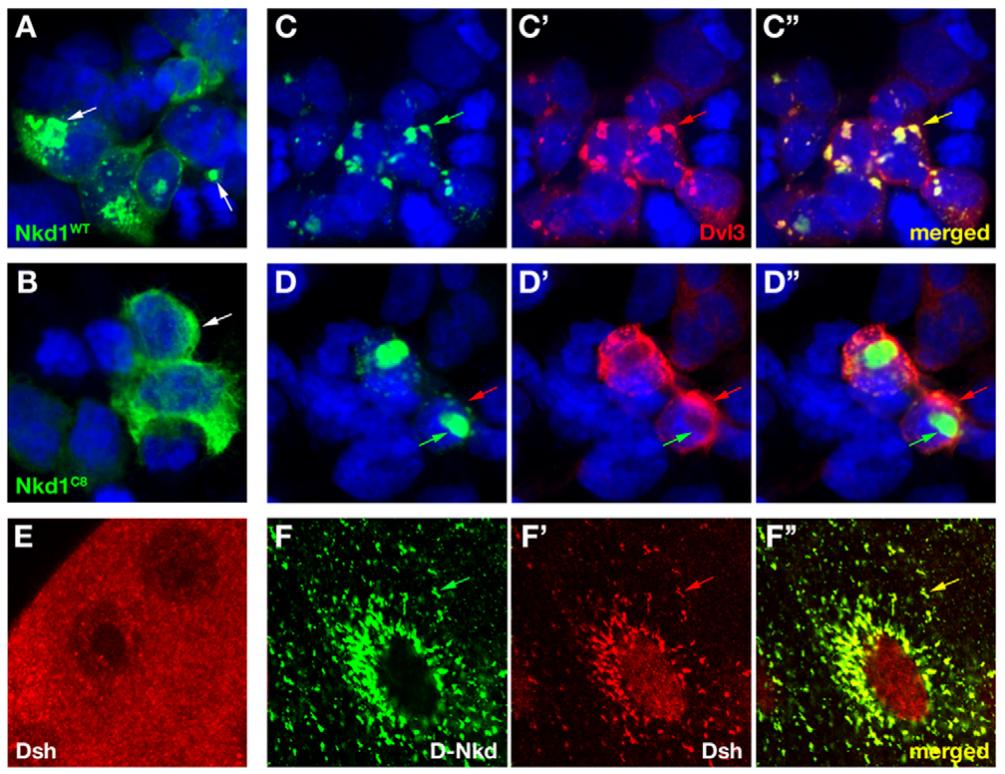
Nkd1 truncation alters subcellular localization and Dvl colocalization. (A, B) HEK-293 cells expressing HA/Flag-tagged Nkd1 (A) or Nkd1^C8^ (B) stained with α-HA (green). Nkd1 accumulates in puncta (arrows), while Nkd1^C8^ is diffusely distributed in the cytoplasm. Nkd1^C6^ distributed in a pattern similar to Nkd1^C8^ (not shown). (C, D) Cells co-expressing Dvl3 and wild-type (C) or C8 (D) Nkd1^GFP^ constructs. Nkd1-GFP (green, C) shows near complete colocalization (arrows) with Dvl3 (red, C′), while Nkd1^C8^-GFP (D) accumulates in cytoplasmic aggregates surrounded by Dvl3 (arrows). Merged images are in C″, D″, DNA is blue in A–D. Similar results were observed in cells coexpressing Nkd1^C8^-GFP and Dvl1 or Dvl2, and in cells expressing Nkd1^C6^-GFP and Dvl1, Dvl2, or Dvl3 (not shown). (E) Salivary gland from wild-type third instar *Drosophila* larva stained with α-Dsh (red) showing diffuse and punctate staining. (F–F″) *A8-Gal4/UAS-Nkd^GFP^* salivary gland stained with α-Dsh and imaged for GFP (F) and Dsh (F′; merged in F″). Fly Nkd^GFP^ relocalizes Dsh to perinuclear aggregates (arrows) similar to those observed with Nkd1^GFP^/Dvl3 in C.

We were unable to detect endogenous Nkd1 in cell lines by immunocytochemistry, so to further investigate the relationship between Nkd1 localization and activity we examined the localization of tagged proteins in HEK-293 cells. Nkd1 localizes in a punctate, predominantly cytoplasmic distribution similar to *Drosophila* Nkd^GFP^ when expressed in fly salivary gland (Fig. 4A, F). In contrast, Nkd1^C6^ and Nkd1^C8^ distribute diffusely in cytoplasm (Fig. 4B and not shown), consistent with their enhanced detergent solubility relative to Nkd1 (Fig. 1E), while Nkd1^R288H^ aggregates like wild-type Nkd1 (not shown).

Dvl proteins localize to dynamic, cytoplasmic and plasma membrane-associated aggregates that have been proposed to amplify Wnt/β-catenin signaling [24]. Consistent with *in vitro* Nkd/Dsh association, expression of Nkd1^GFP^ in HEK-293 cells or fly Nkd^GFP^ in salivary gland gave rise to intracellular aggregates with colocalized Nkd and Dsh/Dvl (Fig. 4C–C″, F–F″, and not shown). In contrast, each Dvl co-synthesized with each truncated Nkd1^GFP^ (C6 or C8) formed aggregates, but colocalization was instead observed at aggregate interfaces, with Dvl aggregates typically surrounding truncated Nkd1 aggregates (Fig. 4D–D″ and not shown). Although the significance of these localizations vis-à-vis Wnt signaling is unclear, the truncated Nkd1 proteins identified in CRC exhibited a reduced ability to colocalize with Dvl proteins as compared to full-length Nkd1.

### Mutant Nkd1 proteins are defective at binding Dvl proteins

**Figure 5:**
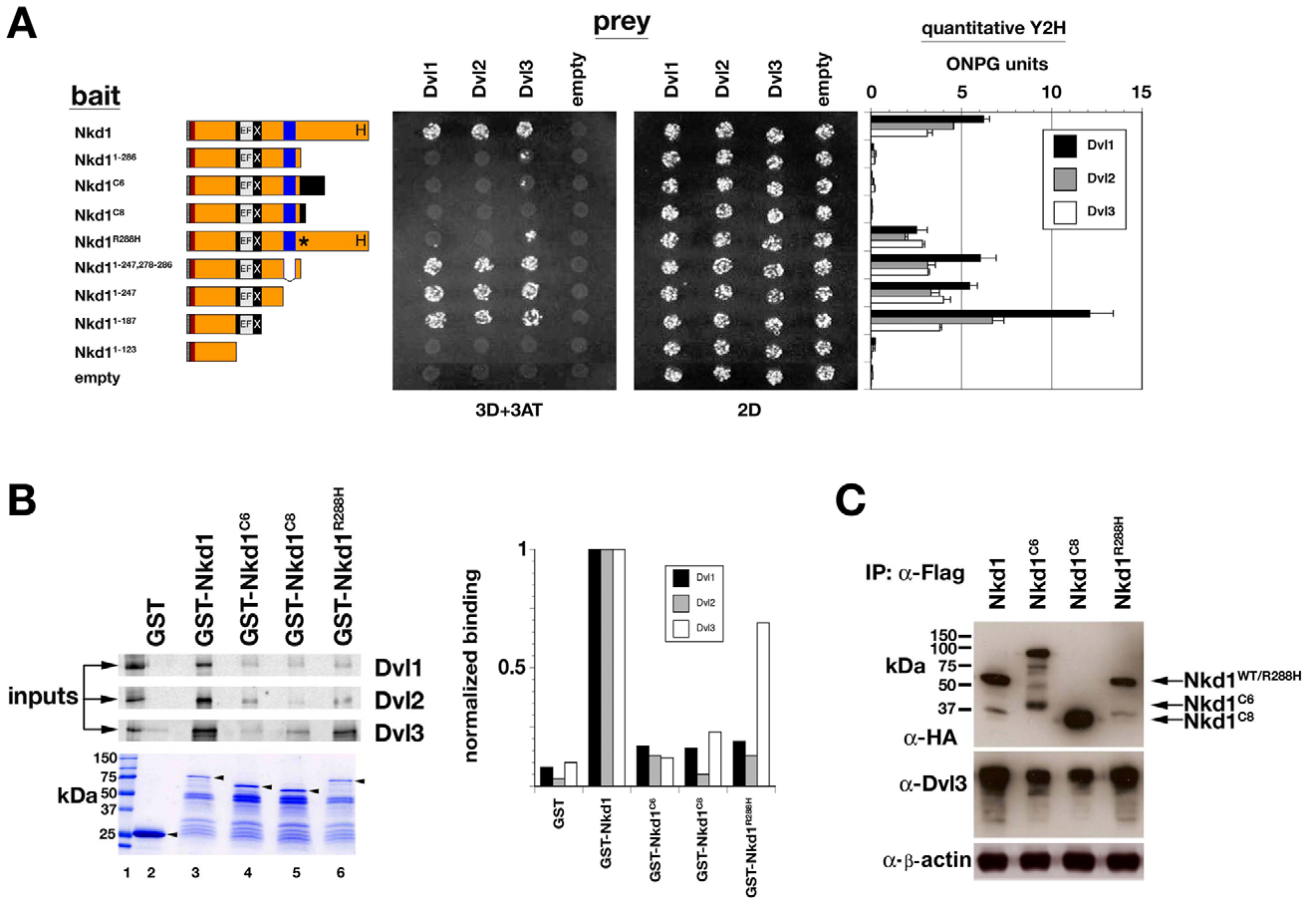
Mutant Nkd1 proteins exhibit reduced Dsh/Dvl associations. (A) Y2H between Nkd1-baits and Dvl-prey assayed for growth on triple dropout (3D+3AT) or double dropout (2D) media. Bar graphs on right show ONPG units from quantitative Y2H assay. Western blot indicates that Nkd1 mutant proteins are of comparable abundance (not shown). (B) GST-pulldown assay of *in vitro* translated, ^35^[S]-labelled Dvl1-3 with each GST-Nkd1 protein. Bar graph is quantitative band intensity. Coomassie-stained gel shows each GST-fusion protein, with arrowheads indicating full-length proteins. (C) Western blots of Flag-IPs from HEK-293 cell extracts with equal amounts of each HA/Flag-tagged Nkd1, and then probed with α-HA (upper) and α-Dvl3 (middle). β-actin is loading control (lower). Note that less Dvl3 is IP'd by Nkd1^C6^ or Nkd1^C8^ as compared to full-length Nkd1. No co-IP was observed between wild-type or mutant Nkd1s and Dvl1 or Dvl2, reminiscent of the lack of stable co-IP between *Drosophila* Nkd and Dsh [13].

Next we investigated the biochemical mechanism of defective Wnt signal inhibition by mutant Nkd1 proteins. The Nkd EFX motif binds the basic/PDZ region of Dsh/Dvl proteins [14]. Surprisingly, each Nkd1 mutant, despite having an intact EFX motif, bound each Dvl protein less than wild-type Nkd1 by yeast-two-hybrid (Y2H) assay (Fig. 5A). Nkd1 truncation at the (C)_7_-tract (Nkd1^1–286^) also reduced Dvl binding (Fig. 5A), indicating that reduced Dvl binding was not due to frameshift-induced unique C-termini in the two truncated mutants. Each truncated Nkd1 protein retains near its C-terminus a 30aa amphipathic α-helical motif that is highly conserved (28/30 aa) in Nkd2 [14]. In fly Nkd, a similarly positioned 30aa motif is critical for function and nuclear localization [25], but the role of the 30aa motif in vertebrate Nkd proteins remains unknown. Deletion of the 30aa motif in Nkd1^1–286^ restored Dvl binding, whereas further deletion of the EFX motif eliminated Dvl binding (Fig. 5A). These data suggest that one function of the vertebrate Nkd 30aa motif is to oppose Nkd1-EFX/Dvl interactions, which is itself apparently opposed by further C-terminal sequence that is deleted in our MSI-CRC tumors.

We confirmed the Y2H results by GST-pulldown and coimmunoprecipitation experiments. As shown in Fig. 5B, each Dvl protein exhibited reduced binding to GST-Nkd1^C6^ and GST-Nkd1^C8^ as compared to wild-type GST-Nkd1, while GST-Nkd1^R288H^ showed reduced associations with Dvl1 and Dvl2, but less so with Dvl3, similar to that seen by Y2H. When expressed in HEK-293 cells, Nkd1^C6^ and Nkd1^C8^ accumulated to higher levels than Nkd1 and Nkd1^R288H^ (not shown); by normalizing input lysates so that equal amounts of wild-type Nkd1 and each mutant Nkd1 protein were immunoprecipitated, we observed a two-fold reduction in the amount of Dvl3 co-immunoprecipitated by Nkd1^C6^ or Nkd1^C8^ as compared to Nkd1 or Nkd1^R288H^ (Fig. 5C). Thus, the *NKD1* mutations reduce Nkd1/Dvl associations *in vitro* and *in vivo*.

### Mutant Nkd1s are defective at altering Dvl levels

**Figure 6:**
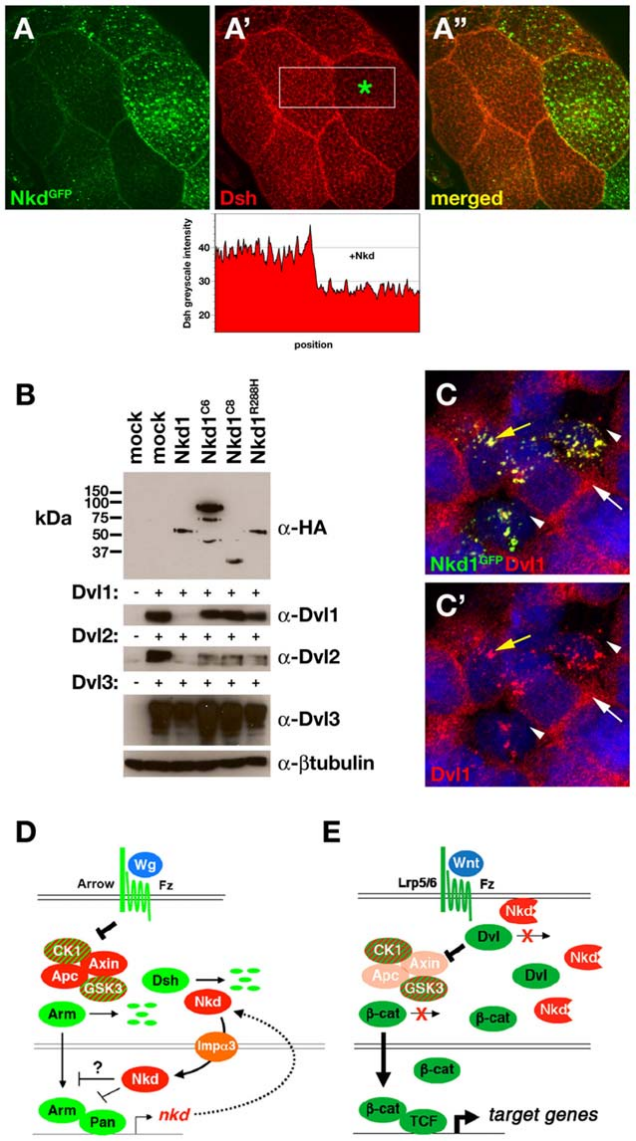
Mutant Nkd1s do not limit the abundance of Dsh/Dvl as well as wild type Nkd1. (A–A″) *71B-Gal4/UAS-Nkd^GFP^* third-instar *Drosophila* salivary gland stained with α-Dsh and imaged for GFP (A) and Dsh (A′) distributions (merged image in A″). Quantitation of Dsh pixel intensity (white box in A′) reveals reduced staining in cell expressing more (right, green asterisk) Nkd^GFP^ than in adjacent cell expressing less Nkd^GFP^. (B) Western blots of HEK-293 cells transfected with indicated Flag/HA-tagged Nkd1 and Dvl1, Dvl2, or Dvl3 constructs probed with α-HA, α-Dvl1-3, and α-βtubulin as a loading control. Each of the Dvl1-3 blots was loaded with equal amounts of extract, as confirmed by probing each blot with α-βtubulin. (C) HEK-293 cells co-expressing Nkd1^GFP^ and Dvl1, stained with α-Dvl1, and imaged for GFP (green), Dvl1 (red), and DNA (blue) showing fine punctate Dvl1 distribution in cells expressing low to absent Nkd1^GFP^ (white arrow). In adjacent cells expressing Nkd1^GFP^, Dvl1 is relocalized to Nkd1^GFP^/Dvl1 aggregates (yellow arrow), with loss of fine punctate Dvl1 staining (arrowheads). (D, E) Models of Nkd function in *Drosophila* (D) and *NKD1*-mutant CRC (E). Double lines: upper = plasma membrane; lower = nuclear membrane. (D) Fly Wg(Wnt) binds Fz/Arrow(Lrp5/6) receptors, which inhibits the Apc/Axin/CK1/GSK3β complex that promotes degradation of Arm(β-catenin). Arm complexes with Pan(TCF) to activate target genes including *nkd*. Nkd promotes Dsh turnover to partially inhibit signaling, and employs the nuclear import factor Imp-α3 to enter the nucleus and further inhibit signaling through unknown mechanisms [31]. (E) In *NKD1*-mutant CRC, the mutant Nkd1 protein no longer binds and promotes Dvl turnover, stabilizing β-catenin and activating TCF-dependent transcription of target genes.

Dvls can be ubiquitinated and degraded by the proteasome, and the binding of Nkd or other Dvl-binding proteins leads to Dvl turnover [17], [26]–[28]. The *NKD1* mutations might therefore compromise the ability of Nkd1 to destabilize Dvls. By expressing *Drosophila* Nkd^GFP^ at different levels in adjacent cells of the third-instar *Drosophila* salivary gland, we consistently observed an inverse relationship between levels of Nkd^GFP^ and Dsh (Fig. 6A–A″). Since *dsh* transcription is not known to be regulated in *Drosophila*, Nkd^GFP^ is likely destabilizing Dsh, as observed when Nkd1 was overexpressed in cultured mammalian cells [17]. Next, we co-transfected wild-type or each mutant Nkd1 with Dvl1, Dvl2, or Dvl3 into HEK-293 cells and examined the levels of each Dvl protein by western blot. As shown in Fig. 6B, Dvl1 and Dvl2, and to a lesser extent Dvl3, were less abundant when co-expressed with wild-type Nkd1 than when expressed alone. Neither empty-vector nor co-expressed GFP affected the levels of each Dvl (not shown). In contrast, the amount of Dvl1 detectable with co-expression of each mutant Nkd1 was similar to the Dvl1-only transfected control (Fig. 6B). Dvl2 levels were partially reduced by each mutant Nkd1, while Dvl3 levels were reduced less by coexpression of either truncated Nkd1 than by wild-type Nkd1. Similar to the inverse relationship between Nkd^GFP^ and Dsh levels in *Drosophila* salivary gland (Fig. 6A–A″), fine punctate Dvl1 immunoreactivity in cells with high levels of Nkd1^GFP^ appeared reduced compared to adjacent cells with lower levels of Nkd1^GFP^ (Fig. 6C,C′). Taken together, our data support the hypothesis that the specific alteration of Nkd1's ability to promote Dvl turnover might activate Wnt/β-catenin signaling during CRC tumor progression.

## Discussion

Mutation of the tumor suppressor *APC* elevates Wnt/β-catenin signaling in the majority of the >1 million new cases of CRC diagnosed annually world-wide [3], [29]. We hypothesized that mutations in other Wnt antagonists might elevate signaling in the subset of CRC, particularly MSI-CRC, without mutations in known Wnt regulators. We report three cancer-associated human *NKD1* mutations that alter Wnt/β-catenin signaling and disrupt Nkd1/Dvl binding. Based on the frequency of *NKD1* mutation (5%) identified in our cohort of MSI-CRCs, we estimate that *NKD1* mutations occur in up to ∼1% of newly diagnosed CRC, or ∼10,000 cases per year.

Since MSI tumors are prone to mutation throughout the genome, the question arises of whether the *NKD1* mutations drive tumor progression or are merely “bystander” mutations. A National Cancer Institute workshop [30] proposed five criteria to distinguish bona-fide target genes from bystander mutations, including a) high mutation frequency, b) biallelic inactivation, c) a role in a growth suppressor pathway, d) inactivation of the same growth suppression pathway in tumors without MSI through mutation in the same gene or in another gene within the same pathway, and e) functional suppressor studies, although the validity of these criteria in evaluating rare or novel driver mutations has been questioned {e.g. [6]}. Our work suggests that the *NKD1* mutations fulfill four of the five criteria – a, c, d, and e. While the frequency of *NKD1* mutation was relatively low compared to that of known Wnt pathway genes, a mutual exclusivity among mutations in *NKD1* and other Wnt pathway genes was statistically significant in tumor samples. The absence of *NKD1* mutations in our sample of tumors without MSI could be due to the rare nature of specific Nkd1 truncation in tumors with intact MMR, or due to our small sample size. However, other genes in the Wnt signaling pathway such as *APC* are frequently mutated, deleted, or methylated in tumors with intact MMR. Biallelic inactivation of *NKD1* was not observed, but the presence of wild type Nkd1 in tumor cell lines, as well as the ability of mutant Nkd1 to stabilize β-catenin, suggests that the *NKD1* mutations might act dominantly (see below). Finally, the Nkd family of proteins inhibits canonical Wnt signaling, and this activity is defective in all three mutant Nkd1 proteins, suggesting that the Nkd1 mutations alter Wnt/β-catenin signaling *in vivo*.

We further demonstrate that Nkd can limit Dsh/Dvl abundance in both mammalian cell culture and fly systems, with fly Nkd additionally having unknown but essential nuclear functions [25], [31] (Fig. 6D). We propose that the mutant human Nkd1 proteins, each with a reduced ability to bind and limit the abundance of Dvls, increase Wnt-dependent Dvl activity, thereby attenuating β-catenin degradation and increasing TCF-dependent transcription of target genes that promote proliferation (Fig. 6E). However, preventing direct Nkd1/Dvl association is apparently insufficient to promote neoplasia, as deletion of the Dvl-binding *Nkd1* EFX motif neither rendered mutant mice susceptible to cancer nor potentiated the frequency of *Apc* mutation-driven intestinal adenomas [32]. Similarly, mice carrying N-terminal truncating *Nkd1* and/or *Nkd2* mutations did not develop spontaneous tumors [33]. Since Nkd is integral to feedback loops in flies and vertebrates [12], [34], we previously hypothesized that in mammals a lack of Nkd activity can be compensated by redundant feedback mechanisms, whereas in flies no such compensation is possible given the absence of genes encoding extracellular Wnt signaling antagonists [33].

The non-random pairing of dissimilar *APC* mutations in tumors {e.g., protein truncation near the mutation cluster region (MCR) with allelic deletion or methylation}, coupled with functional studies of mutant Apc proteins [35], [36], has suggested that the cell must retain some ability to regulate Wnt/β-catenin signaling during tumor progression – the so-called “just right” hypothesis [37]. A consideration of the known roles for Wnt/β-catenin signaling during colorectal carcinoma progression provides some rationale for this hypothesis: in early stages, increased signaling promotes stem cell renewal and alters the migration of crypt epithelial cells [38], whereas later it acts as a switch to regulate epithelial to mesenchymal transitions during invasion and metastasis [39]. Thus, tumor progression might require transient up- or down-regulation of target gene expression depending on mutational load and local environmental conditions. Given that wild-type Nkd1 protein persists in the *NKD1*-mutant cell lines tested, we hypothesize that the mutant Nkd1 proteins - each of which retains multiple functional motifs (N-terminal myristoylation, EFX, and 30 aa motifs) - activate Wnt signaling *in vivo*, perhaps analogous to the manner in which Apc proteins truncated near the MCR activate Wnt signaling [36].

Despite the overwhelming evidence that abnormal Wnt/β-catenin signaling causes cancer, the role of Dsh/Dvl proteins in neoplasia remains obscure. Wnt signaling can promote Dsh/Dvl accumulation [40], and Dvl overexpression can mimic activation of the Wnt/β-catenin signaling axis [41], suggesting that Dvl hyperactivity, like β-catenin stabilization due to mutation, could be a primary cause of elevated Wnt signaling in cancer. Indeed, Dvl amplification and overexpression has been identified in neoplasia {e.g. lung cancer [42]}, but Dvl accumulation in cancer could also be a secondary consequence of unopposed Wnt ligand-driven autoactivation of signaling [43]. Given the crucial roles for Dsh/Dvls in “non-canonical” Wnt pathways that govern planar-cell-polarity and cell migration in vertebrates [44], the *NKD1* mutations might also alter Dvl activity in non-canonical Wnt pathways that control cell polarity or migration during cancer progression. Future experiments will focus on understanding how the mutant Nkd1 proteins alter Wnt signaling during cancer progression *in vivo*.

## Materials and Methods

### Ethics statement

This study was conducted according to the principles expressed in the Declaration of Helsinki. The study was approved by the Institutional Review Board of Mayo Clinic hospitals. All patients provided written informed consent for the collection of samples and subsequent analysis. Frog husbandry, in vitro fertilization, and embryo culture and staging were performed according to standard protocols and all animals were handled in strict accordance with good animal practice as defined by the American Association for Laboratory Animal Science, and the *Xenopus* studies were approved by the Animal Care and Use committee at the University of Pennsylvania.

### CRC cell lines and tumors

DNA from 11 MSI-CRC cell lines (Co115, LS174, Lovo, TC71, HCT15, HCT116, TC7, SW48, RKO, HCT8, and LS411) was provided by R.H.. DNA from 5 CRC cell lines without MSI (SW480 from U. Verma, UTSW; SW837, SW620, HT29, and Caco-2, from ATCC) was isolated as described [8]. Forty primary MSI-CRC and 50 CRC without MSI were collected at Mayo Clinic [8]. Following microdissection of tumor cells from sections of tumor specimens, DNA was extracted using the Easy-DNA™ kit (Invitrogen).

### Mutation detection

All *NKD1* coding exons and intron/exon junctions were PCR-amplified (sequences available upon request). Primer pairs used to amplify the *NKD1* poly(C)_7_ tract: 5′TCTGGGGTATAGCGCAAGC and 5′CTGAGACCTTGGCGATTGG. Methods for PCR, denaturing high-performance liquid chromatography (DHPLC), direct sequencing, and for detecting mutations in *AXIN2*, *TCF7L2*, *CTNNB1*, and *APC* were as described [8], [45].

### Plasmids

*NKD1* cDNA was isolated from a human lung cDNA library (U. Sathyanarayana and A. Gazdar, UT Southwestern) by PCR. All PCR reactions used Pfu polymerase. *NKD1* codons in expression constructs were as follows: Nkd1^WT^ 1–470; Nkd1^C6^ 1–287 followed by out-of-frame sequence for codons 288–345; Nkd1^C8^ 1–287 followed by out-of frame sequence for codons 288–298; Nkd1^R288H^ 1–470 with Arg #288 changed to His. For expression in HEK-293 cells, *NKD1* cDNAs with a 3′ HA-epitope tag (YPYDVPDY) were PCR-amplified and cloned into pCMV-4B (Sigma-Aldrich; 3′ Flag-tag) to generate Nkd1 constructs with C-terminal HA/Flag-tags. For Nkd1^GFP^s, Nkd1 fragments were PCR amplified and cloned into pEGFP-N3 (Clontech). For *Xenopus* injections, each HA/Flag-tagged Nkd1 fragment was subcloned into pCS2+ (http://sitemaker.umich.edu/dlturner.vectors). Human Dvl1, Dvl2, and Dvl3 were expressed via the CMV promoter in pCDNA3 (Invitrogen). For the TOPflash assay, mouse Dvl2 cDNA was PCR-amplified and cloned into pEGFP-N1 (Clontech). For cell proliferation assays, Nkd1 fragments were PCR-amplified and cloned into pBABE-puro. Retroviral transductions were performed as described (http://www.addgene.org/pgvec1?f=c&cmd=findpl&identifier=1764).

### *Xenopus* injections

pCS2+ EGFP, XWnt8, and Nkd1 (wild type/mutant) plasmids were linearized and mRNAs transcribed with SP6 mMessage mMachine (Ambion). Axis duplication assays were performed by injecting 1 pg *XWnt8* +/−250 pg *NKD1* (wild-type or mutant) with 200 pg EGFP (lineage tracer) mRNAs into one ventral blastomere at the 4-cell stage. Embryos were collected at stage 10, and lysates were analyzed by western blot with α-HA (Sigma) at 1∶1,000. The remaining embryos were cultured until stage 35, fixed, and scored for axis phenotype: Single axis: wild-type; Single axis (short A/P): embryos without secondary axes, but with marked reduction in the A/P axis; Partial Axis Duplication: embryos with ectopic trunk structures, but no ectopic head tissues; Full Axis Duplication: embryos bearing ectopic trunk and anterior structures including cement gland and at least one eye or forebrain vesicle.

### TOPflash assay

HEK-293 cells were cultured in DMEM+10% horse serum, 100 u/ml pen-strep, 1 mM sodium pyruvate, 2 mM L-glutamine, and 0.1 mM nonessential amino acids. 5×10^4^ cells were seeded into each well of a 24-well plate and co-transfected with 0.3 µg plasmid including 0.1 µg TOPflash (Upstate Biotechnology) +/−0.1 µg pCMV-Dvl2^GFP^ +/−0.1 µg each pCMV-Nkd1 expression construct or empty vector +1 ng pRL-TK as a control for transfection efficiency. TCF reporter activity was measured 36 hr post-transfection with the Dual-Luciferase Reporter Assay System (Promega), and luciferase activities were measured using a Lumat LB-9507 luminometer (Berthold). Assays were performed in triplicate.

### Detection of endogenous Nkd1 and β-catenin

*Whole cell lysates*: 2×10^6^ cells were harvested with 5 mM EDTA in PBS, pelleted at 300 g for 5 min at 4°C, lysed in NETN-100 lysis buffer (100 mM NaCl, 1 mM EDTA, 20 mM Tris pH 8.0, 0.5% NP-40 containing complete protease inhibitor cocktail (Roche), 10 mM NaF, and 10 mM β-glycerolphosphate), and lysates were centrifuged at 10,000 g for 10 min at 4°C. Protein concentrations were determined by Bradford assay (Bio-Rad). Supernatants were resolved by 10% SDS-PAGE. *Triton X-100 lysates*: 3×10^5^ cells were rinsed in PBS, and incubated with 0.5 ml 50 mM MES buffer, pH 6.8 (2.5 mM EGTA, 5 mM MgCl_2_ and 0.5%Triton X-100) for 3 min at room temperature. Triton X-100-soluble lysates were then aspirated into a fresh tube. The remaining material, scraped into 0.5 ml MES buffer and vortexed for 1 min, constituted the insoluble fraction. 50 µg each extract was resolved by 10% SDS-PAGE. After Hybond membrane (GE Healthcare) transfer, western blot was performed at 4°C overnight with rabbit polyclonal α-Nkd1 (Ab1, Cell Signaling Technology) at 1∶1,000 with 0.1% Triton X-100 and 5% nonfat powdered milk (as blocking agent) in TBS, followed by incubation at room temperature for 1 hr with HRP-conjugated secondary antibodies (Pierce) at 1∶1,000. Signals were visualized by the SuperSignal West Chemiluminescent Substrate kit (Pierce). The specificity of α-Nkd1 antisera was confirmed by western blot of *nkd1*-mutant tissues [33]. For Fig. 3D, HEK-293T cells were transfected with 4 µg each plasmid (wild type or each mutant Nkd1), and 48 hrs later subcellular fractionation was performed as described [46]. Concentrations of extract fractions were determined by BCA Protein Assay Kit (Pierce) and confirmed by immunoblots.

### Cell proliferation assay

2×10^3^ cells/well were plated in triplicate into 96-well plates and infected 2 hrs later with retrovirus expressing wild-type or each mutant Nkd1, or retrovirus control (titers≥10^7^/ml). Medium was replaced with 100 µl fresh medium 24 hrs post infection. Cell numbers were counted using the MTS assay (Promega).

### *In vitro* protein binding assays

Nkd constructs were cloned into the pAS2-1 bait vector, Dvl constructs into the pAct2 prey vector (Clontech). Yeast-two-hybrid assay and yeast extract preparation was as described [47] in the yeast reporter strain Y190 under double dropout (2D: -Leu-Trp) and triple dropout (3D: -Leu-Trp-His) conditions with 50 mM 3-amino-1,2,4-triazole (3-AT) (Sigma) added to suppress growth under 3D. Four independent experiments were performed in triplicate for each plasmid combination.

Lysates containing each GST-Nkd fusion protein were prepared from BL21pLys *E. coli* (Amersham Biosciences). Each lysate was incubated with glutathione-Sepharose-4B beads for 1 hr at 4°C, and then washed 3x with DT80 buffer [47]. ^35^[S]-Methionine-labeled Dvl1, Dvl2, and Dvl3 proteins were synthesized using TNT T7 coupled reticulocyte lysate system (Promega) and incubated with beads for 2 hr at 4°C in DT80 buffer. The beads were washed 4x with DT300 buffer, and labeled proteins were eluted in SDS-PAGE sample buffer, boiled 5 min, and then separated by SDS-PAGE on 8% gels. Gels were vacuum dried, and Dvl1-3 protein bands were quantitated using a Typhoon phosphorimager with ImageQuant software (Molecular Dynamics).

### Nkd/Dsh transfection and immunoprecipitations

2.2×10^6^ HEK-293 cells cultured in DMEM+10%FBS were transfected with 12.5 µg HA/Flag-tagged Nkd1 construct and 12.5 µg Dvl1, Dvl2, or Dvl3 using Transfectamine (Invitrogen). 24 hr post-transfection, cells were harvested with 5 mM EDTA in PBS, pelleted at 300 g for 5 min at 4°C, and then lysed in NETN-100 (described above) for 1 hr on ice. Lysate was centrifuged at 10,000 g for 5 min at 4°C, and extract volumes containing equal amounts of wild-type and each mutant Nkd1 protein (as determined by quantitating a western blot with loaded with equal volumes of total protein extract) were immunoprecipitated (IP'd) using the M2 α-Flag IP kit (Sigma-Aldrich). IPs were eluted with 150 ng/ml 3X-Flag peptide (Sigma-Aldrich) followed by western blot as described above. Antibodies used at 1∶1,000: mouse mAb α-HA HA.11 (Covance); mouse mAb α-Dvl1(3F12; Santa Cruz Biotechnology); rabbit polyclonal α-Dvl2 and α-Dvl3 (#3216, #3218; Cell Signaling Technology). In Fig. 6B, 3.3×10^5^ HEK-293 cells were transfected with 0.2 µg each HA/Flag-tagged Nkd1 and 0.2 µg Dvl1, Dvl2, or Dvl3. 24 hr-post transfection, cells were harvested and whole cell lysates were prepared as described above. α-Dvl Abs were used at 1∶1000; mAb α-βtubulin (TU27, Covance Research Products) 1∶2,500. Control Abs were used as follows: α-βactin (#A5541, Sigma,) 1∶5,000; α-GAPDH (25778, Santa Cruz Biotechnology) 1∶5,000; α-HDAC2 (6296, Santa Cruz Biotechnology) 1∶500.

### Immunofluorescence staining and microscopy

For β-catenin detection, CRC cell lines cultured on 12-well slides (Erie Scientific Co.) were fixed in 4% formaldehyde in PBS for 15 min, permeabilized with 0.2% Triton X-100 (1xPBS) for 5 min, and blocked in 3% BSA (1xPBS) for 30 min. The cells were incubated with mouse monoclonal α-β-catenin antibody (BD Transduction Laboratories) followed by Alexa 594-conjugated donkey anti-mouse IgG (Molecular Probes). Images were acquired with an Olympus BX41.

For detection of transfected Nkd/Dsh, 4×10^4^ HEK-293 cells in each well of poly-D-lysine–coated glass 8-chambered slides (BD Biosciences) were transfected with 0.2 µg each tagged Nkd1 plasmid construct +/−0.2 µg Dvl1, Dvl2, or Dvl3 plasmid using Lipofectamine 2000 (Invitrogen). 36 hrs post-transfection, slides were fixed with 4% paraformaldehyde for 10 min and permeabilized with 0.2% Triton X-100 in PBS. After blocking with 10% BSA (Sigma-Aldrich) in PBS for 1 hr, cells were incubated with each α-Dvl Ab at 4°C overnight. Antibody dilutions: α-Dvl1 (3F12) 1∶100; α-Dvl2 (#3216) 1∶250; α-Dvl3 (#3218) 1∶250; α-HA (HA.11) 1∶1,000. Mouse mAbs were incubated with TRITC-conjugated rabbit α-mouse IgG (Invitrogen) at 1∶200 in 10% BSA/PBS, while rabbit polyclonal Abs were incubated with Rhodamine Red-X conjugated goat α-rabbit IgG (Invitrogen) at 1∶200. Images were acquired on a Nikon-C1 confocal. The specificity of each α-Dvl antisera was verified by lack of staining in untransfected HEK-293 cells, and in Dvl-transfected cells incubated minus each Dvl antibody but incubated with the fluorescent secondary Ab.

### *Drosophila* experiments

Salivary glands from wild-type, *A8-Gal4/UAS-Nkd^GFP^*, or *71B-Gal4/UASNkd^GFP^* third instar larvae were fixed for 10 min in PBS+4% paraformaldehyde at 4°C, then stained with α-Dsh at 1∶100 and Alexa-588 conjugated α-rabbit secondary Ab at 1∶500 (Jackson Immunoresearch). Confocal images were acquired as described [25]. Image pixel intensities were analyzed with ImageJ (NIH).

## References

[1] Barker N, Clevers H (2006). Mining the Wnt pathway for cancer therapeutics.. Nat Rev Drug Discov.

[2] Clevers H (2006). Wnt/β-Catenin Signaling in Development and Disease.. Cell.

[3] Segditsas S, Tomlinson I (2006). Colorectal cancer and genetic alterations in the Wnt pathway.. Oncogene.

[4] Morin PJ, Sparks AB, Korinek V, Barker N, Clevers H (1997). Activation of β-catenin-Tcf signaling in colon cancer by mutations in *β-catenin* or *APC*.. Science.

[5] Lengauer C, Kinzler KW, Vogelstein B (1997). Genetic instability in colorectal cancers.. Nature.

[6] Duval A, Hamelin R (2002). Mutations at coding repeat sequences in mismatch repair-deficient human cancers: toward a new concept of target genes for instability.. Cancer Res.

[7] Toft NJ, Arends MJ (1998). DNA mismatch repair and colorectal cancer.. J Pathol.

[8] Liu W, Dong X, Mai M, Seelan RS, Taniguchi K (2000). Mutations in *AXIN2* cause colorectal cancer with defective mismatch repair by activating β-catenin/TCF signalling.. Nat Genet.

[9] Duval A, Gayet J, Zhou XP, Iacopetta B, Thomas G (1999). Frequent frameshift mutations of the *TCF-4 (TCF7L2)* gene in colorectal cancers with microsatellite instability.. Cancer Res.

[10] Huang J, Papadopoulos N, McKinley AJ, Farrington SM, Curtis LJ (1996). *APC* mutations in colorectal tumors with mismatch repair deficiency.. Proc Natl Acad Sci U S A.

[11] Thorstensen L, Lind GE, Lovig T, Diep CB, Meling GI (2005). Genetic and epigenetic changes of components affecting the WNT pathway in colorectal carcinomas stratified by microsatellite instability.. Neoplasia.

[12] Zeng W, Wharton KA, Mack JA, Wang K, Gadbaw M (2000). *naked cuticle* encodes an inducible antagonist of Wnt signalling.. Nature.

[13] Rousset R, Mack JA, Wharton KA, Axelrod JD, Cadigan KM (2001). *naked cuticle* targets *dishevelled* to antagonize Wnt signal transduction.. Genes Dev.

[14] Wharton KA, Zimmermann G, Rousset R, Scott MP (2001). Vertebrate proteins related to *Drosophila* Naked Cuticle bind Dishevelled and antagonize Wnt signaling.. Dev Biol.

[15] Katoh M (2001). Molecular cloning, gene structure, and expression analyses of *NKD1* and *NKD2*.. Int J Oncol.

[16] Yan D, Wallingford JB, Sun TQ, Nelson AM, Sakanaka C (2001). Cell autonomous regulation of multiple Dishevelled-dependent pathways by mammalian *Nkd*.. Proc Natl Acad Sci U S A.

[17] Creyghton MP, Roel G, Eichhorn PJ, Hijmans EM, Maurer I (2005). PR72, a novel regulator of Wnt signaling required for Naked cuticle function.. Genes Dev.

[18] Hamada F, Tomoyasu Y, Takatsu Y, Nakamura M, Nagai S (1999). Negative regulation of Wingless signaling by *D-axin*, a *Drosophila* homolog of *axin*.. Science.

[19] McCartney BM, Dierick HA, Kirkpatrick C, Moline MM, Baas A (1999). *Drosophila APC2* is a cytoskeletally-associated protein that regulates wingless signaling in the embryonic epidermis.. J Cell Biol.

[20] Nishisho I, Nakamura Y, Miyoshi Y, Miki Y, Ando H (1991). Mutations of chromosome 5q21 genes in FAP and colorectal cancer patients.. Science.

[21] Li C, Franklin JL, Graves-Deal R, Jerome WG, Cao Z (2004). Myristoylated Naked2 escorts transforming growth factor alpha to the basolateral plasma membrane of polarized epithelial cells.. Proc Natl Acad Sci U S A.

[22] Chan CC, Zhang S, Cagatay T, Wharton KA (2007). Cell-autonomous, myristyl-independent activity of the Drosophila Wnt/Wingless antagonist Naked cuticle (Nkd).. Dev Biol.

[23] Bejsovec A, Wieschaus E (1993). Segment polarity gene interactions modulate epidermal patterning in *Drosophila* embryos.. Development.

[24] Schwarz-Romond T, Fiedler M, Shibata N, Butler PJ, Kikuchi A (2007). The DIX domain of Dishevelled confers Wnt signaling by dynamic polymerization.. Nat Struct Mol Biol.

[25] Waldrop S, Chan C-C, Cagatay T, Zhang S, Rousset R (2006). An unconventional nuclear localization motif is crucial for function of the *Drosophila* Wnt/wingless antagonist Naked cuticle.. Genetics.

[26] Zhang L, Gao X, Wen J, Ning Y, Chen YG (2006). Dapper 1 antagonizes Wnt signaling by promoting dishevelled degradation.. J Biol Chem.

[27] Chan DW, Chan CY, Yam JW, Ching YP, Ng IO (2006). Prickle-1 negatively regulates Wnt/β-catenin pathway by promoting Dishevelled ubiquitination/degradation in liver cancer.. Gastroenterology.

[28] Angers S, Thorpe CJ, Biechele TL, Goldenberg SJ, Zheng N (2006). The KLHL12-Cullin-3 ubiquitin ligase negatively regulates the Wnt-β-catenin pathway by targeting Dishevelled for degradation.. Nat Cell Biol.

[29] Parkin DM, Bray F, Ferlay J, Pisani P (2005). Global cancer statistics, 2002.. CA Cancer J Clin.

[30] Boland CR, Thibodeau SN, Hamilton SR, Sidransky D, Eshleman JR (1998). A National Cancer Institute Workshop on Microsatellite Instability for cancer detection and familial predisposition: development of international criteria for the determination of microsatellite instability in colorectal cancer.. Cancer Res.

[31] Chan CC, Zhang S, Rousset R, Wharton KA (2008). Drosophila Naked cuticle (Nkd) engages the nuclear import adaptor Importin-α3 to antagonize Wnt/β-catenin signaling.. Dev Biol.

[32] Li Q, Ishikawa TO, Miyoshi H, Oshima M, Taketo MM (2005). A targeted mutation of *Nkd1* impairs mouse spermatogenesis.. J Biol Chem.

[33] Zhang S, Cagatay T, Amanai M, Zhang M, Kline J (2007). Viable mice with compound mutations in the Wnt/Dvl pathway antagonists *nkd1* and *nkd2*.. Mol Cell Biol.

[34] Dequeant ML, Glynn E, Gaudenz K, Wahl M, Chen J (2006). A complex oscillating network of signaling genes underlies the mouse segmentation clock.. Science.

[35] Vleminckx K, Wong E, Guger K, Rubinfeld B, Polakis P (1997). Adenomatous polyposis coli tumor suppressor protein has signaling activity in *Xenopus laevis* embryos resulting in the induction of an ectopic dorsoanterior axis.. J Cell Biol.

[36] Takacs CM, Baird JR, Hughes EG, Kent SS, Benchabane H (2008). Dual positive and negative regulation of *wingless* signaling by *adenomatous polyposis coli*.. Science.

[37] Albuquerque C, Breukel C, van der Luijt R, Fidalgo P, Lage P (2002). The ‘just-right’ signaling model: APC somatic mutations are selected based on a specific level of activation of the β-catenin signaling cascade.. Hum Mol Genet.

[38] de Lau W, Barker N, Clevers H (2007). WNT signaling in the normal intestine and colorectal cancer.. Front Biosci.

[39] Hlubek F, Spaderna S, Schmalhofer O, Jung A, Kirchner T (2007). Wnt/FZD signaling and colorectal cancer morphogenesis.. Front Biosci.

[40] Yanagawa S, van Leeuwen F, Wodarz A, Klingensmith J, Nusse R (1995). The dishevelled protein is modified by wingless signaling in *Drosophila*.. Genes Dev.

[41] Sokol SY, Klingensmith J, Perrimon N, Itoh K (1995). Dorsalizing and neuralizing properties of *Xdsh*, a maternally expressed *Xenopus* homolog of *dishevelled*.. Development.

[42] Uematsu K, He B, You L, Xu Z, McCormick F (2003). Activation of the Wnt pathway in non small cell lung cancer: evidence of dishevelled overexpression.. Oncogene.

[43] Bafico A, Liu G, Goldin L, Harris V, Aaronson SA (2004). An autocrine mechanism for constitutive Wnt pathway activation in human cancer cells.. Cancer Cell.

[44] Veeman MT, Axelrod JD, Moon RT (2003). A second canon. Functions and mechanisms of β-catenin-independent Wnt signaling.. Dev Cell.

[45] Powell SM, Petersen GM, Krush AJ, Booker S, Jen J (1993). Molecular diagnosis of familial adenomatous polyposis.. N Engl J Med.

[46] Guo J, Jin G, Meng L, Ma H, Nie D (2004). Subcellullar localization of tumor-associated antigen 3H11Ag.. Biochem Biophys Res Commun.

[47] Rousset R, Wharton KA, Zimmermann G, Scott MP (2002). Zinc-dependent interaction between dishevelled and the *Drosophila* Wnt antagonist naked cuticle.. J Biol Chem.

[48] Gayet J, Zhou XP, Duval A, Rolland S, Hoang JM (2001). Extensive characterization of genetic alterations in a series of human colorectal cancer cell lines.. Oncogene.

